# Applicability of the WHO Maternal Near Miss Criteria in a Low-Resource Setting

**DOI:** 10.1371/journal.pone.0061248

**Published:** 2013-04-16

**Authors:** Ellen Nelissen, Estomih Mduma, Jacqueline Broerse, Hege Ersdal, Bjørg Evjen-Olsen, Jos van Roosmalen, Jelle Stekelenburg

**Affiliations:** 1 Haydom Lutheran Hospital, Mbulu, Manyara, Tanzania; 2 Athena Institute, Faculty of Earth and Life Sciences, VU University Amsterdam, Amsterdam, The Netherlands; 3 Stavanger Acute Medicine Foundation for Education and Research, Stavanger University Hospital, Stavanger, Norway; 4 Centre for International Health, University of Bergen, Bergen, Norway; 5 Department of Obstetrics and Gynaecology, Sørlandet Hospital, Flekkefjord, Norway; 6 Department of Obstetrics, Leiden University Medical Centre, Leiden, The Netherlands; 7 EMGO Institute for Health and Care Research, VU University Medical Centre, Amsterdam, The Netherlands; 8 Department of Obstetrics and Gynaecology, Leeuwarden Medical Centre, Leeuwarden, The Netherlands; University of Vermont College of Medicine, United States of America

## Abstract

**Background:**

Maternal near misses are increasingly used to study quality of obstetric care. Inclusion criteria for the identification of near misses are diverse and studies not comparable. WHO developed universal near miss inclusion criteria in 2009 and these criteria have been validated in Brazil and Canada.

**Objectives:**

To validate and refine the WHO near miss criteria in a low-resource setting.

**Methods:**

A prospective cross-sectional study was performed in a rural referral hospital in Tanzania. From November 2009 until November 2011, all cases of maternal death (MD) and maternal near miss (MNM) were included. For identification of MNM, a local modification of the WHO near miss criteria was used, because most laboratory-based and some management-based criteria could not be applied in this setting. Disease-based criteria were added as they reflect severe maternal morbidity. In the absence of a gold standard for identification of MNM, the clinical WHO criteria were validated for identification of MD.

**Results:**

32 MD and 216 MNM were identified using the locally adapted near miss criteria; case fatality rate (CFR) was 12.9%. WHO near miss criteria identified only 60 MNM (CFR 35.6%). All clinical criteria, 25% of the laboratory-based criteria and 50% of the management-based criteria could be applied. The threshold of five units of blood for identification of MNM led to underreporting of MNM. Clinical criteria showed specificity of 99.5% (95%CI: 99.4%–99.7%) and sensitivity of 100% (95%CI: 91.1%–100%). Some inclusion criteria did not contribute to the identification of cases and therefore may be eligible for removal.

**Conclusion:**

The applicability of the WHO near miss criteria depends on the local context, e.g. level of health care. The clinical criteria showed good validity. Lowering the threshold for blood transfusion from five to two units in settings without blood bank and addition of disease-based criteria in low-resource settings is recommended.

## Introduction

In 2011, 273,465 (uncertainty interval 256,332-291,693) women died worldwide during pregnancy, childbirth or within 42 days after childbirth [1]. The majority of these women die in low-income countries, and sub-Saharan Africa carries the highest burden, with a maternal mortality ratio (MMR) ranging between 169/100,000 live births in Southern sub-Saharan Africa and 478/100,000 live births in West sub-Saharan Africa [1].

Worldwide the numbers of maternal deaths are high, but at hospital level numbers become scarce. It is important to understand the process of care that the patient has undergone in order to improve the quality of care. In this context, near miss events of women who almost died, but survived pregnancy related complications, are increasingly used in order to evaluate the functioning of the health system [2], [3]. Near miss cases represent most of the characteristics of maternal deaths, but occur more often [4], [5]. In addition, auditing near miss cases may be less threatening for the involved healthcare workers, because the cases can also be seen as ‘great saves’.

Identification criteria for maternal near misses or severe acute maternal morbidity were mainly divided into three areas: disease-based, management-based and organ-dysfunction-based criteria [6]. However, since inclusion criteria are not uniform [2], [3], [7] and studies not comparable, the World Health Organization (WHO) developed a new definition of maternal near miss (MNM) and formulated identification criteria for maternal near miss cases in 2009 [8]. A maternal near miss is defined by WHO as a woman who nearly died but survived a complication that occurred during pregnancy, childbirth or within 42 days of termination of pregnancy [8]. The inclusion criteria for a maternal near miss are categorised in three areas: clinical criteria, laboratory-based criteria and management-based criteria. The goal is that these identification criteria may be used in any setting, regardless of the development status. They should be comparable across settings and over time, and there should be a high threshold for identification of cases in order not to overload the health system with extra work [8]. The development of the near miss criteria resulted in 2011 in the WHO near miss approach [9]. This is a guideline for evaluating the quality of care for severe pregnancy complications, based on the concept of criterion-based clinical audit [10].

The WHO laboratory-based and management-based criteria were validated in Brazil and Canada [8], [11], [12]. The results of the pre-validation showed that the WHO near miss criteria could identify all cases of maternal death and almost all cases who experienced organ failure [11].

The aims of this study were 1) to validate the WHO near miss criteria, especially the clinical criteria, in a low-resource setting, and 2) to further refine the WHO near miss criteria. This article reports on the experience of applying the WHO near miss criteria prospectively in a low-resource setting where the burden of maternal mortality and morbidity is high.

## Methods

A prospective cross-sectional study was conducted from November 2009 until November 2011 at Haydom Lutheran Hospital (HLH), a 400-bed referral hospital in Northern Tanzania. The hospital is located in an isolated, rural area, 300 km southwest from the nearest city, Arusha. Extrapolating from the 2002 census, the immediate catchment area covered a population of 327,000 in 2010. The greater reference area covered a population of approximately 2,200,000 [13]. The hospital provides free reproductive and child health services, and comprehensive emergency obstetric care, including ambulance and radio service. Furthermore there is an Intensive Care Unit (ICU) with 24-hours medical supervision and facilities for mechanical ventilation.

### Inclusion Criteria

All maternal deaths and maternal near misses that were admitted to HLH were prospectively included in the study during the above-mentioned period. In this study, a maternal death (MD) is defined as the death of a woman while pregnant or within 42 days of termination of pregnancy, from any cause. For the identification of maternal near misses, we intended to use the WHO near miss criteria, but not all WHO criteria were applicable in this low-resource setting. Therefore, a local modification of the criteria for use in Haydom was made (table 1). The definitions used for the Haydom near miss clinical criteria were the same as the definitions used for the WHO near miss clinical criteria. All clinical criteria could be applied in HLH. In the group of laboratory-based criteria, only oxygen saturation and measurement of platelets could be used. The other six laboratory-based criteria, PaO2, creatinine, bilirubin, pH, lactate, and ketoacids in the urine were not available in the laboratory and could therefore not be measured. These were removed from the list of inclusion criteria. In the category management-based criteria, three out of six criteria could be used in HLH (intubation and ventilation, cardio-pulmonary resuscitation (CPR) and hysterectomy). The three management-based criteria that could not be used were use of continuous vasoactive drugs (not available), renal dialysis (not available), and transfusion of five or more units of blood. The threshold for transfusion of five units of blood or more was reduced to at least one unit of blood, as blood is very scarce in this setting and there is no functioning blood bank. Women in need of blood are dependent on family members who serve as blood donors. As a result, transfusion of even one unit of blood may be a true indicator of severe maternal morbidity in HLH. For the same reason, admission to ICU was added to the management-based criteria. Women who require intensive care suffer from illnesses, which may be classified as severe maternal morbidity in the context of HLH. Uterine rupture, eclampsia and sepsis are common causes of maternal mortality [14]. Therefore, the authors unanimously decided to add the diagnoses of uterine rupture, eclampsia and sepsis to the inclusion criteria, as they reflect severe maternal complications and were not covered by the WHO inclusion criteria for near miss. We decided to not include severe postpartum haemorrhage as a separate disease-based criterion, as it was already captured by the inclusion criteria “shock” and “use of blood products”. Data were collected with the local “Haydom near miss criteria”. Afterwards, the WHO near miss criteria were applied to the Haydom data set and compared with the Haydom near miss criteria.

**Table 1:** WHO near miss criteria adapted to the local context of HLH.

WHO near miss criteria [8]	Haydom near miss criteria
**Clinical criteria**	
Acute cyanosis	Acute cyanosis
Gasping	Gasping
Respiratory rate >40 or <6/min	Respiratory rate >40 or <6/min
Shock	Shock^a^
Oliguria non responsive to fluids or diuretics	Oliguria non responsive to fluids or diuretics^b^
Failure to form clots	Failure to form clots^c^
Loss of consciousness lasting >12 h	Loss of consciousness lasting >12 h^d^
Cardiac arrest	Cardiac arrest^e^
Stroke	Stroke^f^
Uncontrollable fit/total paralysis	Uncontrollable fit/total paralysis^g^
Jaundice in the presence of pre-eclampsia	Jaundice in the presence of pre-eclampsia^h^
**Laboratory-based criteria**	
Oxygen saturation <90% for ≥60 minutes	Oxygen saturation <90% for ≥60 minutes
PaO2/FiO2<200 mmHg	
Creatinine ≥300µmol/l or ≥3.5 mg/dL	
Bilirubin >100 µmol/l or >6.0 mg/dL	
pH <7.1	
Lactate >5 mEq/mL	
Acute thrombocytopenia (<50,000 platelets/ml)	Acute thrombocytopenia (<50,000 platelets/ml)
Loss of consciousness and ketoacids in urine	
**Management-based criteria**	
	Admission to intensive care unit
Use of continuous vasoactive drugs	
Hysterectomy following infection or haemorrhage	Hysterectomy following infection or haemorrhage
Transfusion of ≥5 units of blood	Transfusion of ≥1 unit of blood
Intubation and ventilation for ≥60 minutes not related to anaesthesia	Intubation and ventilation for ≥60 minutes not related to anaesthesia
Dialysis for acute renal failure	
Cardio-pulmonary resuscitation	Cardio-pulmonary resuscitation
**Severe maternal complications**	
	Eclampsia^i^
	Sepsis or severe systemic infection^j^
	Uterine rupture^k^

a Shock is defined as a persistent severe hypotension, defined as a systolic blood pressure <90 mmHg for 60 min with a pulse rate of ≥120/min despite aggressive fluid replacement (>2L).

b Oliguria is defined as an urinary output <30 ml/hour for 4 hours or <400 ml/24 hr.

c Failure to form clots is defined as the absence of clotting from the IV site after 7–10 minutes.

d Unconsciousness/coma lasting >12 hours is defined as a profound alteration of mental state that involves complete or near-complete lack of responsiveness to external stimuli or Glasgow Coma Scale <10.

e Cardiac arrest is defined as loss of consciousness and absence of pulse or heart beat.

f Stroke is defined as a neurological deficit of cerebrovascular cause that persists ≥24 hours, or is interrupted by death within 24 hours.

g Uncontrollable fit is a condition in which the brain is in state of continuous seizure.

h Pre-eclampsia: the presence of hypertension associated with proteinuria. Hypertension is defined as a blood pressure ≥140 mmHg (systolic) or ≥90 mmHg (diastolic). Proteinuria is defined as excretion of ≥300 mg protein/24 hr or 300 mg protein/litre urine or ≥1+ on a dipstick.

i Eclampsia is defined as the presence of hypertension associated with proteinuria and fits. Hypertension is defined as a blood pressure ≥140 mmHg (systolic) or ≥90 mmHg (diastolic). Proteinuria is defined as excretion of ≥300 mg protein/24 hr or 300 mg protein/litre urine or ≥1+ on a dipstick.

j Sepsis is defined as a clinical sign of infection and 3 of the following: temp>38°C or <36°C, respiration rate >20/min, pulse rate >90/min, WBC >12.

k Uterine rupture is defined as the complete rupture of a uterus during labour.

### Data Collection and Quality Assessment

Cases were identified on a daily basis by either the first author (EN) or by one of the two trained research assistants (nurse-midwives). This was achieved through daily participation in the morning report and daily visits to the maternity ward, ICU and the internal medicine ward. When the inclusion criteria were met, a structured questionnaire was filled out after discharge or death of the woman. Data were obtained from hospital files. The completed questionnaires were checked by a second person on missing data or discrepancies. If needed, a copy of the hospital file was checked to validate the recordings. Variables that were collected for this study were information on presence of an inclusion criterion and final outcome (MNM or MD). Furthermore, measurements to identify clinical and laboratory-based criteria were noted (physical examination, vital signs, urinary output, oxygen saturation and full blood count). All data were double entered and cross-checked in Epidata [15].

### Validation

Previous validation of the WHO near miss criteria was done with the Sequential Organ Failure Assessment (SOFA) score as gold standard for the definition of MNM [11]. Six variables were used to determine the SOFA score: measurement of PaO2 or FiO2, platelet count, measurement of bilirubin, hypotension (and the use of continuous vasoactive drugs), the Glasgow Coma Score, and measurement of creatinine or urinary output [16]. In this low-resource setting we were able to collect only three of the six variables that were used to determine the SOFA score (platelet count, the Glasgow Coma Score, urinary output). Therefore the SOFA score could not be used as gold standard. In addition, it is questionable to use the SOFA score for validation because the WHO criteria were derived from it. Instead, we validated the clinical WHO near miss criteria for the identification of maternal deaths, as maternal deaths should be comparable to maternal near misses, except for the vital status [5].

### Refining the Near Miss Criteria

In order to refine the WHO near miss criteria, and thereby improve the applicability in practice, we performed a stepwise elimination process, to determine which criteria were most important. The inclusion criteria were ranked from most to least frequently used. Subsequently, the most frequently used inclusion criterion was excluded, and all cases with this inclusion criterion were (temporarily) removed from the database. Thereafter, frequencies of inclusion criteria were calculated again for the remaining cases and a new ranking was created. This stepwise elimination process helped us to rank the inclusion criteria according to importance (frequency of use).

### Statistical Analysis

Data analysis consisted of frequencies of the use of inclusion criteria, the presence of physical examination and measurement of vital signs, urinary output, oxygen saturation and full blood count. Validity of the (clinical) WHO near miss criteria and Haydom near miss criteria was assessed by calculating sensitivity, specificity, positive predictive value and negative predictive value against outcome (maternal death), among all women who delivered during the study period. All results are reported as number (n) and frequency (%). Analysis was performed using SPSS Statistics, version 20 (SPSS Inc. Chicago, Illinois).

### Ethical Clearance

The study was performed in full accordance with the guidelines for medical research of the Helsinki declaration of 1975, as revised in 2008. Ethical approval was obtained from the Tanzanian National Institute for Medical Research (NIMR) (reference NIMR/HQ/R.8a/Vol.IX/1247), the Tanzania Commission for Science and Technology (COSTECH) (reference 2012-56-NA-2011-201), and from the VU university medical centre (VUmc), the Netherlands (reference 2011/389). As stated by the VU university medical centre, the study does not fall within the scope of the Medical Research Involving Human Subjects Act, and formal approval was not needed. Data were collected and extracted from patient records without any identification of the subject. Questionnaires were filled in after discharge or death and therefore study inclusion did not have effect on the treatment. Considering this approach and the statement of the VUmc, individually obtained informed consent was not required.

## Results

According to the local Haydom near miss criteria, 248 women with life-threatening conditions were included in the two-year study period: 216 MNM and 32 MD. When the WHO near miss criteria were applied to this dataset, 92 women with life-threatening conditions remained, of which 60 MNM and 32 MD (table 2). In the two-year study period 9,471 deliveries and 9,136 live births occurred at HLH, resulting in a severe maternal outcome ratio of 27.1 per 1,000 live births. Case fatality rate (CFR) for cases identified with the Haydom near miss criteria was 12.9%, whereas CFR for cases identified with the WHO criteria was 35.6%.

**Table 2:** Use of near miss inclusion criteria.

	Haydom (n = 248)	WHO (n = 92)	Excluded (n)
**MNM**	216	60	156
**MD**	32	32	0
**Clinical criteria**			
Acute cyanosis	–	–	–
Gasping	15	15	0
Respiratory rate >40 or <6/min	10	10	0
Shock	51	51	0
Oliguria non responsive to fluids or diuretics	4	4	0
Failure to form clots	3	3	0
Loss of consciousness lasting >12 h	16	16	0
Cardiac arrest	26	26	0
Stroke	4	4	0
Uncontrollable fit/total paralysis	3	3	0
Jaundice in the presence of pre-eclampsia	3	3	0
**Laboratory based criteria**			
Oxygen saturation <90% for ≥60 minutes	17	17	0
Acute thrombocytopenia	12	12	0
**Management based criteria**			
Admission to intensive care unit	91	63	28
Hysterectomy following infection or haemorrhage	16	16	0
Use of blood products	184	58	126
Intubation and ventilation for ≥60 minutes not related to anaesthesia	15	15	0
Cardio-pulmonary resuscitation	19	19	0
**Severe maternal complications**			
Eclampsia	15	5	10
Sepsis	30	20	10
Uterine rupture	20	13	7

Women can have more than one inclusion criterion.

**Figure 1:**
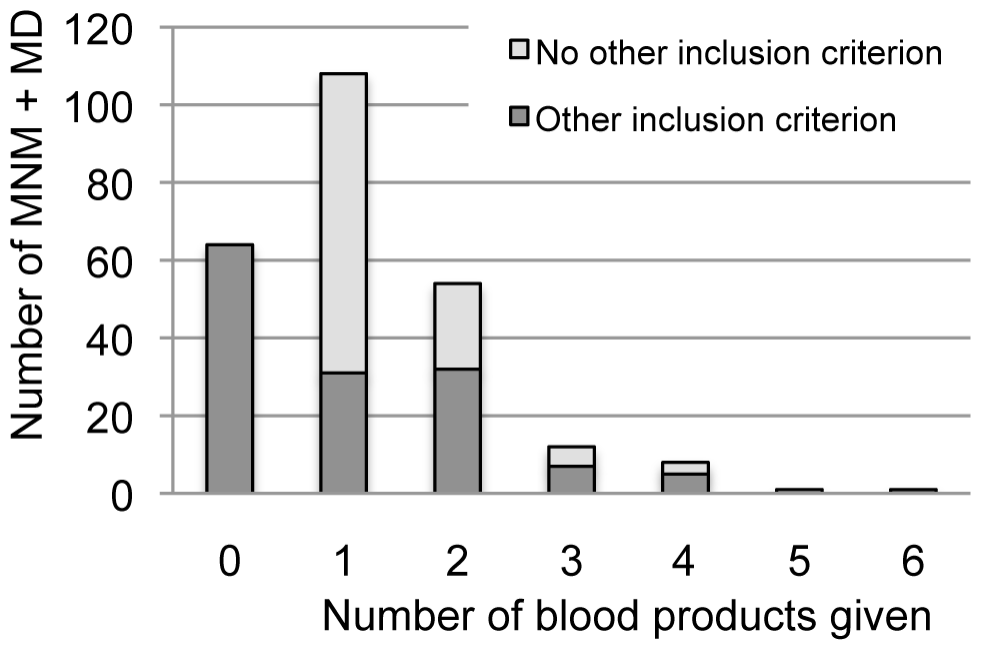
Threshold for blood transfusion.

The difference in the number of near misses can be attributed largely to the different threshold for the transfusion of blood. For the Haydom criteria one unit was chosen, as compared to five units for the WHO criteria. Of 248 women that were included with the Haydom near miss criteria, 184 women received one unit of blood or more, compared to 58 of the 92 women selected with the WHO criteria. Two of the 58 cases had transfusion of five units or more. Figure 1 shows the number of blood products given, and whether there were other inclusion criteria or not. One hundred eight women received one unit of blood. Of these women, 77 women did not have another inclusion criterion. Fifty-four women received two units of blood, of which 22 women did not have another inclusion criterion. Increasing the threshold to five units of blood led to the inclusion of two cases only. Excluding the 77 women who received only one unit of blood, would lead to a rise in CFR from 12.9% to 22.6% (32/171).

Other differences in the number of near misses could be attributed to the inclusion of all cases with eclampsia, sepsis, uterine rupture and admission to ICU (table 2). Women with these conditions were treated appropriately and did not reach a stage of (multiple) organ failure.

Measurements to identify clinical and laboratory-based criteria were insufficient: 91.5% (n = 227) of 248 women with life-threatening conditions had physical examination on arrival. Blood pressure was measured in 74% (n = 183); pulse rate was noted in 68% (n = 169) and temperature in 65% (n = 161). Urinary output was measured in 10% (n = 25) of all cases. Oxygen saturation on admission was only noted in 4.8% (n = 10) of all women with life-threatening conditions. Full blood count (including platelet count) was taken in 90.1% (n = 155) of 172 women that were ill on arrival.

The clinical WHO near miss criteria were validated for identifying maternal deaths (table 3). Sensitivity was 100% (95%CI: 91.1%–100%), specificity was 99.5% (95%CI: 99.4%–99.7%), positive predictive value was 41.6% (95%CI: 31.1%–52.8%) and negative predictive value was 100% (95%CI: 100%–100%), among all women who delivered during the study period. In addition, all WHO near miss criteria that could be applied in this setting were validated and show similar sensitivity (100%, 95%CI: 91.1%–100%) and specificity (99.4%, 95%CI: 99.2%–99.5%). The positive predictive value of all criteria combined is lower than the clinical criteria alone (34.8%, 95%CI: 25.6%–44.9%), and the negative predictive value is equal (100%, 95%CI: 100%–100%). Lastly, the Haydom near miss criteria were validated. Sensitivity is 100% (95%CI: 91.1%–100%), specificity is 97.7% (95%CI: 97.4%–98.0%), positive predictive value is 12.9% (95%CI: 9.2%–17.5%) and negative predictive value is 100% (95%CI: 99.9%–100%). If the threshold for blood transfusion would be raised to two units of blood or more, sensitivity would remain at 100% (95%CI: 91.1–100%), specificity would increase slightly (98.5%, 95%CI: 98.3%–98.8%), positive predictive value would increase to 18.7% (95%CI: 13.4%–25.1%), and negative predictive value would remain at 100% (95%CI: 100%–100%).

**Table 3:** Validity of the WHO and Haydom near miss criteria among all women.

	Maternal Outcome = Death			
**WHO clinical criteria**		Positive	Negative	Total
	Positive	32	45	77
	Negative	0	9,394	9,394
	Total	32	9,439	9,471
**WHO near miss criteria**		Positive	Negative	Total
	Positive	32	60	92
	Negative	0	9,379	9,379
	Total	32	9,439	9,471
**Haydom near miss criteria**		Positive	Negative	Total
	Positive	32	216	248
	Negative	0	9,223	9,223
	Total	32	9,439	9,471

**Validity WHO clinical criteria: Sensitivity:** 100%; 95%CI [91.1%–100%], **Specificity:** 99.5%; 95%CI [99.4%–99.7%], **Positive predictive value:** 41.6%; 95%CI [31.1%–52.8%], **Negative predictive value:** 100%; 95%CI [100%–100%].

**Validity WHO near miss criteria: Sensitivity:** 100%; 95%CI [91.1%–100%], **Specificity:** 99.4%; 95%CI [99.2%–99.5%], **Positive predictive value:** 34.8%; 95%CI [25.6%–44.9%], **Negative predictive value:** 100%; 95%CI [100%–100%].

**Validity Haydom near miss criteria: Sensitivity:** 100%; 95%CI [91.1%–100%], **Specificity:** 97.7%; 95%CI [97.4%–98.0%].

**Positive predictive value:** 12.9%; 95%CI [9.2%–17.5%], **Negative predictive value:** 100%; 95%CI [99.9%–100%].

Table 4 presents the contribution per WHO inclusion criterion using a stepwise elimination process, as described in the methods section. The inclusion criterion that was used most frequently was shock. Ninety-two MNM and MD were included with the WHO criteria, of which 51 cases had shock as inclusion criterion. After excluding all women with shock, 41 MNM and MD remained. Frequencies of the remaining inclusion criteria were calculated and now “loss of consciousness lasting >12 h” was most frequently used (n = 13). This stepwise elimination process was continued until all inclusion criteria were excluded actively or eliminated passively. The inclusion criteria that led independently to the identification of MNM and MD cases were shock (n = 51), loss of consciousness lasting >12 h (n = 13), cardiac arrest (n = 8), hysterectomy (n = 8), acute thrombocytopenia (n = 4), intubation and ventilation for ≥60 minutes not related to anaesthesia (n = 2), oxygen saturation <90% for ≥60 minutes (n = 1), respiratory rate >40 or <6/min (n = 1), oliguria non responsive to fluids or diuretics (n = 1), failure to form clots (n = 1), jaundice in the presence of pre-eclampsia (n = 1) and transfusion of five units of blood or more (n = 1). Inclusion criteria that did not have an independent contribution to identification of cases (but only in combination with other inclusion criteria) were: CPR, gasping, stroke, uncontrollable fit and acute cyanosis.

**Table 4:** Stepwise elimination process of the WHO near miss criteria.

	MNM+MD n = 92	STEP 1 n = 41	STEP 2 n = 28	STEP 3 n = 20	STEP 4 n = 12	STEP 5 n = 8	STEP 6 n = 6	STEP 7 n = 0
**Shock**	51 (55.4%)	**EXCL**	–	–	–	–	–	–
**Cardiac arrest**	26 (28.3%)	9 (22.0%)	8 (28.6%)	**EXCL**	–	–	–	–
Cardio-pulmonary resuscitation (CPR)	19 (20.7%)	7 (17.1%)	6 (21.4%)	–	–	–	–	–
**Oxygen saturation <90% for ≥60 min**	17 (18.5%)	8 (19.5%)	5 (17.9%)	2 (10.0%)	1 (8.3%)	1 (12.5%)	1 (16.7%)	**EXCL**
**Hysterectomy**	16 (17.4%)	8 (19.5%)	8 (28.6%)	8 (40.0%)	**EXCL**	–	–	–
**Loss of consciousness lasting >12** **h**	16 (17.4%)	13 (31.7%)	**EXCL**	–	–	–	–	–
Gasping	15 (16.3%)	5 (12.2%)	4 (14.3%)	–	–	–	–	–
**Intubation and ventilation for ≥60 minutes not related to anaesthesia**	15 (16.3%)	6 (14.6%)	5 (17.9%)	2 (10.0%)	2 (16.7%)	2 (25.0%)	**EXCL**	–
**Acute thrombocytopenia**	12 (13.0%)	5 (12.2%)	4 (14.3%)	4 (20.0%)	4 (33.3%)	**EXCL**	–	–
**Respiratory rate >40 or <6/min**	10 (10.9%)	7 (17.0%)	5 (17.9%)	3 (15.0%)	3 (25.0%)	1 (12.5%)	1 (16.7%)	**EXCL**
**Oliguria non responsive to fluids or diuretics**	4 (4.3%)	3 (7.3%)	1 (3.6%)	1 (5.0%)	1 (8.3%)	1 (12.5%)	1 (16.7%)	**EXCL**
Stroke	4 (4.3%)	3 (7.3%)	1 (3.6%)	–	–	–	–	–
**Failure to form clots**	3 (3.3%)	1 (2.4%)	1 (3.6%)	1 (5.0%)	1 (8.3%)	1 (12.5%)	1 (16.7%)	**EXCL**
**Jaundice in the presence of pre-eclampsia**	3 (3.3%)	2 (4.9%)	1 (3.6%)	1 (5.0%)	1 (8.3%)	1 (12.5%)	1 (16.7%)	**EXCL**
Uncontrollable fit/total paralysis	3 (3.3%)	3 (7.3%)	1 (3.6%)	–	–	–	–	–
**Transfusion of blood ≥5 units**	2 (2.2%)	1 (2.4%)	1 (3.6%)	1 (5.0%)	1 (8.3%)	1 (12.5%)	1 (16.7%)	**EXCL**
Acute cyanosis	–	–	–	–	–	–	–	–

**EXCL**: Excluded. **Bold:** Criteria that have an independent contribution to inclusion of cases. **Step 2:** Two inclusion criteria have n = 8. Cardiac arrest is excluded because it was the second most frequent used inclusion criterion from start. **Step 7:** Six cases are left with six corresponding, independently used inclusion criteria (oxygen saturation, respiratory rate >40 or <6/min, oliguria non responsive to fluids or diuretics, failure to form clots, jaundice in the presence of pre-eclampsia and transfusion of blood ≥5 units). They are excluded simultaneously for space reasons.

When this stepwise elimination process was repeated for the Haydom near miss criteria, the most frequently used inclusion criteria were: use of blood products (n = 184), admission to ICU (n = 41), sepsis (n = 7), eclampsia (n = 6), and uterine rupture (n = 5). The following criteria were not used: CPR, oxygen saturation <90% for ≥60 min, loss of consciousness lasting >12 h, gasping, intubation and ventilation for ≥60 min not related to anaesthesia, acute thrombocytopenia, respiratory rate >40 or <6/min, stroke, jaundice in the presence of pre-eclampsia, failure to form clots, uncontrollable fit and acute cyanosis.

## Discussion

In this paper we report on the applicability and validation of the WHO near miss criteria in a 2-year prospective cross-sectional study in the low-resource setting of a rural referral hospital in Tanzania.

The results show that all clinical criteria could be used in this setting. Experience at HLH, however, indicates that it is not easy to recognise clinical criteria. In maternity ward ten nurse-midwives are available each day, divided over three shifts, to take care of 60 in-patients and an average of 15 deliveries per day. Nurse-midwives are the sentinel persons that should identify clinical signs of deterioration of a patient. With this shortage of health care personnel, clinical signs of deterioration may go unnoticed and this could lead to underreporting of maternal near misses.

Only 25% of all laboratory-based criteria could be used in HLH. Oxygen saturation measurement in our setting is only available in the ICU, and thus has only been measured in 4.8% of all cases. Although the laboratory-based criteria could be used in settings in Brazil [11], [12], [17], the use of these criteria may not be feasible in many health institutions in low-income countries due to the unavailability of sophisticated laboratory measurements. Therefore, most studies in sub-Saharan Africa used disease-based or management-based criteria that do not require a sophisticated laboratory [7], [18], [19], except for one study conducted in South Africa that used organ-dysfunction based criteria [20].

The three management-based criteria that could be used at Haydom were easy to identify and therefore used as an inclusion criterion. Because HLH does not have a well-stocked blood bank, the transfusion of five or more units of blood was considered very unlikely and occurred in only two cases. One woman received one unit of allogeneic blood and five units of auto-transfusion, following ruptured ectopic pregnancy. The other woman was diagnosed with molar pregnancy and received two units of blood in another hospital, before being referred to HLH where she received another three units of blood. Figure 1 shows that for HLH the optimal threshold may be set at two units of blood or more. When the threshold was set at two units, 77 women that only had one blood transfusion as inclusion criterion would not have been included. Women in this group were mainly diagnosed with abortion-related complications, antepartum haemorrhage, anaemia in pregnancy and postpartum haemorrhage, and were not considered critically ill. Despite the scarcity of blood, some women received one unit of blood transfusion without a proper indication. There were no maternal deaths in this group of 77 women.

Few studies used the WHO near miss criteria for retrospective identification of near misses [11], [12], [17], [21]. Morse et al. [17] showed how the number of near misses altered when different inclusion criteria were used in a regional referral hospital in Brazil: using disease-based criteria they included 87 MNM, using organ-dysfunction-based criteria they included 14 MNM and using the WHO near miss criteria only 10 MNM were left. Like in our study, using the WHO near miss criteria resulted in a very high threshold for identification of cases. In two other settings in Brazil the WHO near miss criteria were validated [11], [12]. Cecatti et al. used the SOFA score as gold standard for the identification of near misses [16]. Souza et al. validated the WHO near miss criteria for identifying maternal deaths. The results of both validation studies are similar to ours. In a recent study in Malawi, like in our study, the application of organ-dysfunction-based criteria underestimated severe maternal morbidity, because of absence of disease-based criteria in the WHO near miss approach [21].

In our study several inclusion criteria were detected, which did not identify near miss cases independently. These criteria are eligible for removal from the WHO near miss criteria. This will refine the WHO near miss approach and improve its applicability. This is in concordance with Morse et al., who found that only 12 out of 25 WHO near miss criteria contributed to the identification of near misses [17]. Criteria that were not used resemble criteria that were not used in our study (acute cyanosis, gasping, uncontrollable fit and CPR).

All studies showed that the applicability of the WHO near miss criteria depends on the local context and the local availability of resources, e.g. level of health care. Therefore we suggest different criteria that account for different levels of health care. For example, in a district hospital in a low-resource setting, sophisticated laboratory-based criteria are better not used. Even then local adaptation might be needed, as in our setting an advanced laboratory allowed platelet count, which is not necessary the case in other district hospitals.

Most studies in low-resource settings used disease-based criteria, like eclampsia, sepsis and uterine rupture, to identify near miss cases. Excluding these cases leads to underestimation of maternal near miss cases and therefore we recommend these criteria to be added to the WHO near miss approach, despite the fact that definitions of disease-based criteria may vary among settings.

A limitation of our study is that data collection was only done in wards where pregnant women were mostly admitted. It might be possible that we missed cases that were admitted to other wards. However, HLH policy is that all pregnant women are admitted to maternity ward, and therefore the number of cases that might have been missed will be negligible.

Poor documentation has negative impact on the identification of inclusion criteria. Human resource shortage and low educational levels of staff may affect the quality of documentation and therefore may negatively interfere with case identification and data collection.

We realize that our study is a hospital-based study. Many women who suffer from severe maternal morbidity in the district do not reach the hospital.

### Conclusion

To our knowledge, this is the first study in which the WHO near miss criteria were used and validated prospectively in a rural referral hospital in a low-resource setting.

The applicability of the WHO near miss criteria depends on the local context. In our setting, clinical criteria could be applied and show good sensitivity and specificity. However, sophisticated laboratory and some management-based criteria could not be used. We would recommend lowering the threshold for blood transfusion from five to two units in settings where there is no blood bank. Furthermore, in low-resource settings disease-based criteria should be added to the WHO near miss criteria as they reflect severe maternal morbidity.

Future research should focus on validation of the WHO near miss criteria across multiple low-income countries [22], and clinical criteria should be specifically validated for the identification of near misses.
